# Fracture resistance of direct versus CAD-CAM PMMA provisional restorations: An *in vitro* study

**DOI:** 10.6026/973206300213395

**Published:** 2025-09-30

**Authors:** Varsha Mangtani, Subhash Sonkesriya, Alka Gupta, Harsh Chansoria, Anuja Tripathi, Shivani Gupta

**Affiliations:** 1 Department of Prosthodontics and Crown & Bridge, Government College of Dentistry, Indore, Madhya Pradesh, India; 2 Department of Orthodontics and Dentofacial Orthopaedics, Sri Dharmasthala Manjunatheshwara College of Dental Sciences, Dharwad, Karnataka, India

**Keywords:** CAD-CAM, fracture resistance, PMMA, provisional restoration, temporary crowns

## Abstract

The fracture resistance of polymethyl methacrylate (PMMA)-based provisional crowns fabricated using two techniques: conventional direct and Computer-Aided Design/Computer-Aided Manufacturing (CAD-CAM) milling is of interest to dentist. Hence, sixty extracted premolar teeth were restored and tested under simulated occlusal forces. Data showed that CAD-CAM milled restorations exhibited significantly higher fracture resistance than conventionally fabricated crowns. The study shows the mechanical superiority and clinical reliability of CAD-CAM technology. Thus, we show support its use for improved provisional restoration outcomes.

## Background:

Provisional restorations play a vital role in restorative dentistry, serving both functional and aesthetic purposes during the interim period between tooth preparation and placement of the definitive restoration [1]. These temporary prostheses protect the underlying tooth structure, restore masticatory function, and maintain esthetics and occlusion [2]. Traditionally, provisional restorations have been fabricated directly using materials such as polymethyl methacrylate (PMMA), which remains a popular choice due to its ease of manipulation, good aesthetics, and cost-effectiveness [3]. However, advancements in digital technology particularly Computer-Aided Design and Computer-Aided Manufacturing (CAD-CAM) have transformed the approach to fabricating temporary restorations by enhancing both precision and efficiency [4]. Despite its widespread use, the conventional direct technique for PMMA provisional restorations is associated with several limitations, including inconsistent material thickness, internal porosities, and suboptimal marginal adaptation. As this method is largely operator-dependent, the quality and success of the restoration can vary significantly depending on the clinician's skill level [5]. In contrast, CAD-CAM technology offers improved accuracy, reproducibility, and customization. After digital design, the provisional restoration is milled from standardized PMMA blocks using computer-controlled systems, which enhances material consistency and mechanical strength while ensuring a more precise fit [6, 7].

In clinical practice, provisional restorations are exposed to considerable occlusal forces during functional activities such as chewing and speaking. Therefore, they must possess adequate fracture resistance to endure this stress without failure [8]. Premature fracture of temporary restorations can lead to discomfort, functional limitations, and increased time and cost for both the clinician and the patient. A comparative evaluation of the fracture resistance of PMMA-based temporary restorations fabricated using traditional direct techniques versus CAD-CAM methods is essential to determine the most reliable and clinically effective approach [9]. Although conventional techniques remain widely accepted and are considered clinically safe, there is a growing need for comprehensive, controlled investigations to understand better the mechanical performance of provisional restorations produced through different fabrication methods, especially with PMMA materials [10]. Therefore, it is of interest to assess and compare the durability of direct and CAD-CAM fabricated PMMA provisional restorations under fracture load conditions. By systematically evaluating these techniques, this research seeks to identify the approach that offers superior strength and longevity.

## Methodology:

The present study is an *in vitro* investigation aimed at evaluating and comparing the fracture resistance of provisional restorations fabricated using two different techniques: the conventional direct technique and the CAD-CAM milling technique. This is a prospective, experimental, comparative, quantitative, and *in-vitro* study. The study was conducted at the Government College of Dentistry, Indore, Madhya Pradesh, and fracture resistance testing was performed at the KAILTECH Test & Research Centre Pvt. Ltd, Indore and Madhya Pradesh. Prior to beginning the investigation, institutional ethical committee approval was obtained. The materials and methods used in this study include sixty extracted maxillary first and second premolar teeth, which were selected for the study. These teeth were intact and free of caries, abrasion, erosion, or restoration, and were stored in distilled water at room temperature after cleaning. The materials tested included a chemically activated PMMA resin powder-liquid system (UNIFAST TM III) from GC Corporation, Tokyo, Japan, and CAD/CAM-milled PMMA (Telio CAD) from Ivoclar Vivadent AG, Schaan, Liechtenstein. The equipment used included a Universal Testing Machine (Deepak Polyplast Model No. DUTT-101), sample design software (STL file) NX (Unigraphics), CAD software Exocad (Germany), and an extraoral scanner (Auto Scan-DS-EX Pro from Shining 3D, Hangzhou, China). Other materials for preparation included Flexceed polyvinylsiloxane impression material, Templute™ non-eugenol-based temporary luting cement, Ultra Rock Brown Die Stone, and a Shofu crown and bridge tooth preparation kit. The specimens were divided into two groups: Group I (Conventional Provisionalization) and Group II (CAD/CAM Milled Provisionalization), with 30 samples in each group. The specimens were mounted individually in specific plaster moulds using the Ney surveyor, ensuring that the long axis of the tooth was parallel to the mould. Teeth were prepared using the Shofu crown and bridge kit, following standard tooth preparation protocols, with labial shoulder and palatal chamfer finish lines for porcelain-fused-to-metal crowns. For Group I, provisional crowns were made using the direct conventional technique. A putty index was fabricated before tooth preparation, and the provisional material (Unifast TM III) was mixed and placed into the putty index, which was then seated over the mounted tooth model. Parallel vertical lines were marked on the putty index and the model for alignment. After excess material was removed, the provisional restoration was finished and polished using an acrylic finishing and polishing kit. For Group II, an impression was taken to create a stone model, followed by a final impression with polyvinylsiloxane material and pouring the impression with Ultra Rock Brown Die Stone. The stone model was scanned using an extraoral scanner, and the digital model was converted to an STL file. Using Exocad software, provisional crowns were designed, and the STL files were produced. These files were transferred to the milling machine for wet milling.

After the milling process, the sprue was removed, and the crowns were polished. The provisional crowns were then cemented using Templute™ non-eugenol temporary luting cement, and any excess cement was removed after the initial set. The final step involved mechanical testing of the specimens using the universal testing machine. A uniaxial force was applied to each crown at a 135° angle to the tooth's long axis, simulating occlusal loading conditions. The fracture resistance was measured by recording the maximum load at which the crown fractured in Newtons (N). The results provided insight into the fracture resistance of the two different provisional restoration techniques. The data obtained from the study were subjected to statistical analysis with the assistance of a statistician. The data were systematically compiled and organized into a master table, which was then subdivided and presented as individual tables, along with accompanying graphs. Statistical procedures were carried out in two steps: data compilation and presentation, followed by statistical analysis. The data were entered into an Excel sheet and analyzed using SPSS version 21.0. Descriptive statistics were performed, and the comparison of fracture resistance was done using the independent/unpaired Student t-test. The statistical analysis involved the use of several formulae. The mean was calculated by dividing the sum of the individual data points by the number of observations. The standard deviation (SD) measures the variability of the data set around the mean. In contrast, the standard error of the mean (SEM) indicated the variability of the sample mean from the population mean. The student t-test was used to compare the means of two independent groups, and the significance level was determined using the p-value. A p-value greater than 0.05 indicated that the difference was not significant, while p-values less than 0.05, 0.01, and 0.001 indicated varying degrees of significance. The null hypothesis assumed that there was no significant difference in fracture resistance between the two techniques, while the alternative hypothesis claimed a considerable difference. The observations from the study were then presented in tables, such as the fracture resistance of Group I (provisional crown fabricated by the direct conventional technique). The data collected for the study were analyzed with the assistance of a statistician to ensure precise interpretation and presentation. The analysis was carried out in two stages: data compilation and statistical analysis. The data was systematically organized into a master table, subdivided meaningfully, and presented in individual tables with corresponding graphs. The data were entered into Excel and analyzed using SPSS (Statistical Package for the Social Sciences) version 21.0 (IBM, Chicago). Descriptive statistics were used to summarize the data, and the comparison of shear bond strength was performed using the independent/unpaired Student t-test. Statistical procedures included calculating the mean and SD to measure central tendency and variability, respectively, as well as the standard error of the mean to assess the precision of the sample mean. For comparing the two groups, the Student t-test was employed, assuming that the data were normally distributed with equal variances. The p-value was used to determine the level of significance, where a p-value greater than 0.05 was considered not significant, p < 0.05 indicated significance at the 95% confidence level, p < 0.01 signified high significance at the 99% confidence level, and p < 0.001 was regarded as very highly significant at the 99.9% confidence level. The null hypothesis stated that there would be no significant difference in fracture resistance between PMMA-based provisional crowns fabricated by the Direct Conventional technique and the CAD/CAM milled technique. In contrast, the alternative hypothesis proposed that a significant difference would be observed.

## Results:

Sixty extracted maxillary first and second premolar teeth were used for the study. The teeth were selected based on specific criteria such as being intact and free from caries, abrasion, erosion, or restoration. Following cleaning, the teeth were stored in distilled water at room temperature. The specimens were divided into two groups: Group I (Conventional Provisionalization) and Group II (CAD/CAM Milled Provisionalization), with 30 samples in each group. The fracture resistance testing was conducted by applying uniaxial force to each crown at a 135° angle to the tooth's long axis, simulating occlusal loading conditions. The force required to fracture each crown was recorded in Newtons (N), providing the primary data for the study. The fracture resistance of the provisional crowns fabricated by the direct conventional technique (Group I) was found to range from a minimum of 305 N to a maximum of 425 N, with the mean fracture resistance of 384.03 N and a SD of 34.06 N. In contrast, the fracture resistance of provisional crowns fabricated using the CAD/CAM milling technique (Group II) ranged from 840 N to 950 N, with a significantly higher mean fracture resistance of 906.03 N and a SD of 30.73 N. These results clearly demonstrate that crowns fabricated using the CAD/CAM technique has a significantly higher fracture resistance compared to those manufactured using the conventional method. Statistical analysis of the data revealed a significant difference in fracture resistance between the two groups. An independent samples t-test was performed to compare the means of fracture resistance for the two techniques. The results of the t-test showed a highly significant difference (p-value < 0.05), supporting the rejection of the null hypothesis, which suggested no significant difference in fracture resistance between the two techniques. Levene's test for equality of variances indicated that the variances were significantly different, further corroborating the statistically significant difference between the groups.

The t-test statistic value of -62.32 with a p-value < 0.05 suggests that the difference in fracture resistance is substantial. The comparison of fracture resistance between the two groups is further illustrated by the data in Table 1 (see PDF), where the mean fracture resistance for Group I (Direct Conventional) was 384.03 N. For Group II (CAD/CAM), it was 906.03 N. The greater fracture resistance in Group II can be attributed to the more precise and controlled fabrication process offered by CAD/CAM technology, which ensures a more consistent fit and material strength. In contrast, the direct conventional technique, while effective, demonstrated more variability in the fracture resistance values, as shown by the higher SD in Group I. Graphical representations of the data, such as the line diagram (Figure 1 - see PDF) and bar chart (Figure 2 - see PDF), visually emphasize the significant difference in mean fracture resistance between the two groups. These graphs clearly demonstrate that CAD/CAM-fabricated crowns exhibit higher and more consistent fracture resistance compared to crowns fabricated by the direct conventional method. The table above shows a comparison of the mean Fracture Resistance of both groups. The mean ± SD fracture resistance in Group I and Group II was 384.0333 ± 34.05724 N and 906.0333 ± 30.73074 N, respectively. The minimum and maximum fracture resistance observed in Group I was 305.00 N and 425.00 N, respectively. The minimum and maximum fracture resistance observed in Group II was 840.00 N and 950.00 N, respectively. The mean fracture resistance of PMMA-based Provisional crown fabricated by CAD/ CAM milling technique (906.0333) was significantly higher than the mean Fracture Resistance of PMMA-based Provisional crown fabricated by direct conventional technique (384.0333). P-value <0.05 was considered statistically significant. The results of the independent samples t-test, which were used to compare the fracture resistance between the two groups, are shown in Table 2 (see PDF). The t-test statistic value of -62.32 with a p-value < 0.05 suggests a significant difference in fracture resistance between Group I (Conventional Provisionalization) and Group II (CAD/CAM Milled Provisionalization). Levene's test for equality of variances, shown in Table 2 (see PDF), indicated that the variances were significantly different, further supporting the conclusion of a statistically significant difference between the two groups. Additionally, Table 3 (see PDF) provides a detailed breakdown of the group statistics. It shows that both groups had 30 samples each. The mean fracture resistance for Group I (Conventional) was 384.03 N (SD = 34.06 N), and for Group II (CAD/CAM), the mean was 906.03 N (SD = 30.73 N). The standard error of the mean (SEM) for both groups was also provided in Table 3 (see PDF), which reflects the precision of the calculated means.

## Test:

Independent samples t-test/student's t-test was done to compare the difference in fracture resistance values between groups since the data has mean values more than twice that of the SD, thereby the parametric test (t-test) was preferred for analysing the data.

## Inference:

The test shows a statistically significant difference (P<0.05) in mean fracture resistance between the groups owing to the greater fracture resistance in the Provisional crown fabricated by the CAD/ CAM milling technique group than that of the Provisional crown fabricated by the direct conventional technique group. This rejects the null hypothesis that "there is no significant difference in fracture resistance of PMMA-based provisional crowns fabricated by Direct Conventional and CAD/CAM milled digital technique".

## Discussion:

Temporary restorations play a crucial role in restorative dentistry, significantly contributing to both the functional and aesthetic aspects of dental treatment. Their role becomes particularly critical during the interval between tooth preparation and the placement of the definitive restoration [11]. Provisional restorations serve as temporary replacements for extracted or missing teeth, preserving the integrity of the underlying tooth structure and maintaining proper function. Traditionally, these restorations were fabricated using conventional materials such PMMA, which has long been favored for its ease of handling, aesthetic appeal, and satisfactory adhesion to dental structures [5]. Recent advancements in dental technology most notably the introduction CAD-CAM have transformed the fabrication of even temporary restorations. CAD-CAM systems offer considerable advantages over conventional techniques, including increased efficiency, precision, and standardization of restorations [12]. Despite the widespread use of PMMA in provisional restorations, conventional direct fabrication techniques present several challenges. These include inconsistent thickness, internal porosities, and poor marginal adaptation, which are inherent to the manual nature of the process. Such limitations can compromise the long-term success of the restoration, increasing the risk of premature failure. Moreover, the quality of the restoration is often dependent on the clinician's experience and technique, leading to variability in outcomes [13, 14]. In contrast, CAD-CAM technology enhances the quality of temporary restorations through digital design and computer-controlled milling.

This approach ensures greater accuracy, repeatability, and material uniformity. Milling high-performance PMMA blocks also enables improved mechanical properties and an optimal marginal fit, providing better control over the restoration's outcome [4]. Temporary restorations are subjected to considerable mechanical stress from functional activities such as mastication and speech. Therefore, fracture resistance is a critical requirement. A restoration that fails prematurely compromises clinical outcomes and imposes additional costs and inconvenience for both patients and practitioners. As such, comparing the fracture resistance of PMMA-based provisional restorations fabricated using conventional direct methods versus those fabricated using CAD-CAM technology is essential to determine the superior approach [15]. Previous studies have highlighted notable differences in the performance of these two methods. For example, research by Rayyan (2015) suggests that CAD-CAM fabricated restorations exhibit enhanced fracture resistance due to superior material homogeneity and precise fit [16]. Nonetheless, traditional methods remain clinically acceptable in many scenarios. However, further well-controlled and standardised research is needed to comprehensively evaluate the mechanical performance of PMMA restorations fabricated using different techniques.

According to Shekhar *et al.* (2025) [17] Temporary restorations play a crucial role in restorative dentistry, significantly contributing to both the functional and aesthetic aspects of dental treatment. Their role becomes particularly critical during the interval between tooth preparation and the placement of the definitive restoration. Alam *et al.* (2022) [18] described provisional restorations serve as temporary replacements for extracted or missing teeth, preserving the integrity of the underlying tooth structure and maintaining proper function. Traditionally, these restorations were fabricated using conventional materials such as PMMA, which has long been favored for its ease of handling, aesthetic appeal, and satisfactory adhesion to dental structure. Recent advancements in dental technology, most notably the introduction of CAD-CAM systems, have transformed the fabrication of even temporary restorations. CAD-CAM systems offer considerable advantages over conventional techniques, including increased efficiency, precision, and standardization of restorations [19].

## Conclusion:

PMMA provisional restorations made with CAD/CAM milling is significantly less likely to break than those made with the traditional method. CAD/CAM technology extends the lifespan of products, but it's particularly beneficial for items that will be used for an extended period. If one only needs something for a short time, traditional methods are still the best alternative because they are less expensive. Thus, we show the importance of selecting the appropriate technique for each patient, tailored to their specific needs and the unique circumstances of the clinic.
